# Seasonal variability of serum 25-hydroxyvitamin D on multiple sclerosis onset

**DOI:** 10.1038/s41598-021-00344-0

**Published:** 2021-10-25

**Authors:** Pernilla Stridh, Ingrid Kockum, Jesse Huang

**Affiliations:** 1 Center of Molecular Medicine, Karolinska University Hospital, Solna, Sweden; 2 Department of Clinical Neuroscience, Karolinska Institutet, Solna, Sweden

**Keywords:** Neuroimmunology, Risk factors, Multiple sclerosis

## Abstract

Vitamin D deficiency is associated with an increased risk of multiple sclerosis (MS). However, its effect on the age of disease onset remains unclear. This study examines the relationship between serum 25-hydroxyvitamin D (25(OH)D) levels and age of first symptom onset among recently diagnosed MS patients. Serum 25(OH)D was measured from forty MS patients sampled near disease onset. After correcting seasonal variability, the association between 25(OH)D levels, along with other clinical measures such as IgG index, and age at MS onset was examined using multivariable linear regression. Serum 25(OH)D was not correlated with age at onset (*P* > 0.5). We observed bias among previously reported associations between 25(OH)D and MS disease measures resulting from non-random distribution of sampling by season. After correcting for seasonal 25(OH)D and other clinical measures, only CSF IgG index remained significantly associated with age at disease onset (β = − 5.35, *P* = 0.028). In summary, we observed no association between age at onset and serum 25(OH)D levels but observed a negative correlation with CSF IgG index, although this will require further investigation.

## Introduction

Vitamin D is an essential modulator for normal immune function, suppressing inflammation and facilitating immune tolerance^1^. Long-term deficiencies often due to inadequate sun exposure and dietary supplementation have been associated with increased risk of autoimmunity and infection^2^. Low sun exposure has also been associated with an increased risk of multiple sclerosis (MS)^3,4^, a chronic neuroinflammatory disease of the central nervous system. Geographic gradients in MS prevalence, corresponding with latitude and ultraviolet radiation (UVR) levels, has been consistently reported in previous studies^5–7^. Emphasis on early childhood exposures indicates vitamin D is likely involved in the early stages of disease development^4,8^; however, its direct influence on the age at onset or the incidence of pediatric-onset disease remains unclear^4,9,10^. This study investigates the association between vitamin D and age at first symptom onset among recently diagnosed MS patients while also examining effects from seasonal variability using data from Soilu-Hänninen et al.^11^.

## Material and methods

The study cohort and all sampling and analysis protocols have been previously described^11^. In summary, forty MS patients were enrolled from two Finnish neurology clinics between 2000 and 2003, with serum sampled at or near disease onset. Serum 25 hydroxyvitamin D (25(OH)D, nmol/L), a stable precursor commonly used to assess overall vitamin D deficiency, was determined using a commercial I25 radioimmunoassay. Cerebrospinal fluid (CSF) was sampled to determine the CSF IgG index and the presence of oligoclonal bands (OCB). Summary statistics of the study cohort are provided in Table 1.

**Table 1:** Summary characteristics of MS patients.

Variable	Mean ± SD [range]/n (%)
N	40
+ Relapsing-remitting disease	38 (95%)
+ Primary progressive disease	2 (5%)
Female	32 (80%)
Age (years)	36.5 ± 9.0 [18–53]
Age at first symptom (years)	33.8 ± 9.2 [18–53]
Age at diagnosis (years)	36.7 ± 9.1 [18–53]
Disease duration (years)	2.6 ± 3.5 [0–13]
Disease duration, ≤ 1 year	24 (60%)
EDSS ≥ 2	14 (35%)
OCB presence	32 (82.1%)
CSF IgG Index	1.15 ± 0.76 [0.40–4.82]
Serum 25(OH)D level (nmol/L)	49.6 ± 19.4 [17.0–95.0]
Serum 25(OH)D level (adj)* (nmol/L)	47.8 ± 17.7 [4.4–77.6]

*Adj. serum 25(OH)D levels adjusted for seasonal variation.

Serum 25(OH)D levels naturally oscillate due to seasonal variation in sun/UV-B exposure, an important source for vitamin D production in addition to diet and supplementation. Levels tend to peak in the summer and drop in the winter. As previously detailed^8^, 25(OH)D levels were adjusted for seasonal variability using a periodic regression model of sin(2πx/12) + cos(2πx/12), with “x” defined by the month of sampling. Residuals were added to the mean of the model to calculate the season-adjusted 25(OH)D level.

Associations were analyzed using either a Student t-test or a multivariable linear regression model adjusting for sex, age at sampling, and other relevant disease characteristics. The expanded disability status scale (EDSS), assessed at the time of sampling, was dichotomized by ≥ 2, corresponding to at least minimal disability in one functional system^12^. All statistical analyses were performed using R v.4.0.3 (Vienna, Austria).

### Ethics approval

Data for this study was obtained from a publicly available dataset by Soilu-Hänninen et al.^11^, which was approved by the joint Commission on Ethics of the Turku University and the Turku University Central Hospital. Ethical approval for the original study was given by the Joint Commission on Ethics of the Turku University and the Turku University Central Hospital, and the resulting data have already been made publicly available.

## Results

**Figure 1:**
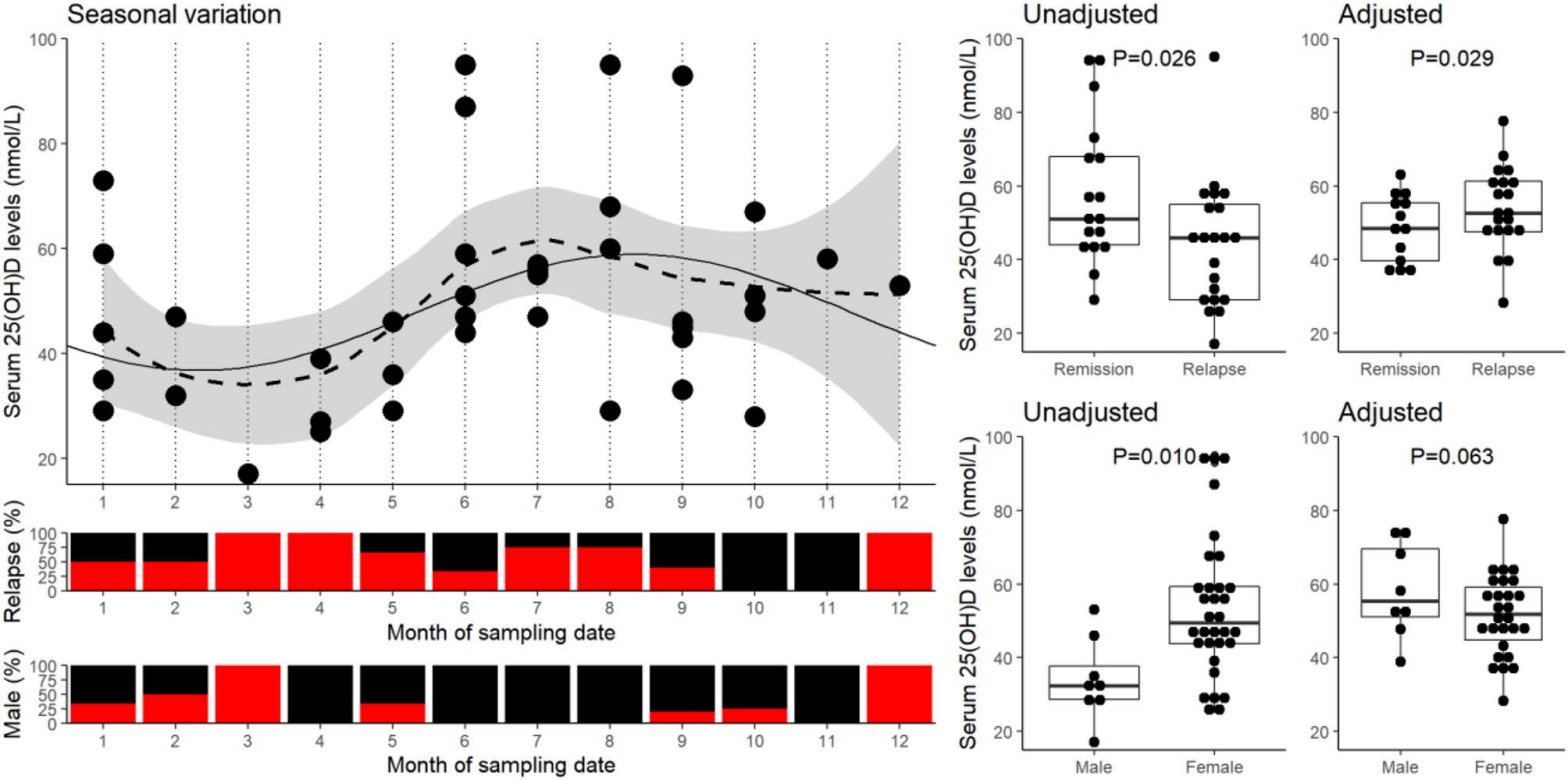
Seasonal variation of serum 25(OH)D levels and its effect on the sampling of relapse/remission patients. The scatter-plot (top-left panel) illustrates the seasonal variation in serum 25(OH)D levels among all MS patients by sampling month and accompanied by the proportion sampled during relapse and of male sex for each month below (bottom-left panels). A smoothed fitted line (dotted, loess method) with 95% confidence interval (grey interval) are plotted along with fitted periodic regression model of sin(2πx/12) + cos(2πx/12) (solid). The distribution of both unadjusted and seasonal variation adjusted serum 25(OH)D levels are illustrated on the right panels (dot plots) along the reported significance between groups as determined by Student t-test.

Figure 1 shows the periodic seasonal variability in serum 25(OH)D levels, with the highest levels observed in the summer and lowest in late winter.

There is a higher proportion of MS patients sampled during relapse between March and May. The risk of sampling a patient undergoing relapse within the period as determined by Fischer’s exact test is OR = 6.132 (95% confidence interval = 0.63–312, *P* = 0.10). Furthermore, this period corresponds with a naturally lower serum 25(OH)D, which explains the previous observation of lower serum 25(OH)D levels among those sampled during relapse compared to remission (β = − 13.9, SE = 6.0, *P* = 0.026)^11^. Following correction of seasonal variation (Fig. 1, right panel), serum 25(OH)D levels among MS patients sampled at relapse were higher than at remission (β = 12.0, SE = 5.3, *P* = 0.029).

A similar effect was observed when comparing 25(OH)D levels by sex. There was a higher proportion of females between June and August (*P* = 0.03) and a moderately higher proportion of males in the winter between December and March (*P* = 0.059). This also results in a switch in effect (uncorrected: β = 19.3, SE = 7.1, *P* = 0.01), with females having lower 25(OH)D than males (corrected: β = − 13.0, SE = 6.8, *P* = 0.06).

Serum 25(OH)D concentration at sampling was not associated with age at onset (*P* > 0.5), even after correcting for sex and other disease characteristics (Table 2). Similarly, Pearson and Spearman correlation coefficients were only ρ = 0.02 and ρ = 0.08, respectively. However, CSF IgG index was negatively correlated with age at onset (*P* < 0.03), and after adjusting for age, sex, and other disease characteristics, IgG index was also positively associated with serum 25(OH)D levels (β = 9.2, SE = 4.0, *P* = 0.03).

**Table 2:** Multivariable linear regression of age at MS symptom onset on serum 25(OH)D levels adjusted for season of sampling and disease characteristics.

Variables	Univariable			Multivariable*		
	β	SE	*P*	β	SE	*P*
Age	0.95	0.06	8.4E−18	–	–	–
Sex, female	3.67	3.65	0.32	6.39	4.23	0.14
Serum 25(OH)D—Unadj	0.05	0.08	0.52	–	–	–
Serum 25(OH)D—Adj	0.01	0.08	0.91	0.06	0.10	0.56
Relapsing activity	0.88	3.05	0.77	− 0.77	3.41	0.82
OCB (+)	− 4.19	3.85	0.28	1.02	4.32	0.81
CSF IgG index	− **4.79**	**1.80**	**0.011**	− **5.35**	**2.31**	**0.028**
EDSS ≥ 2	− 0.13	3.10	0.96	0.72	3.32	0.83

Associations reaching statistical significance (*P* < 0.05) are highlighted in bold.

*All the variables listed were included in the final model.

## Discussion

Our findings did not indicate an association between serum 25(OH)D levels and age at MS onset, consistent with previous studies^4,13–15^. Although this may partly be due to a limited sample size, a multicenter Australian cohort of 213 MS cases observed a similar lack of association^13^. On the contrary, Skalli and colleagues have observed a negative correlation between serum vitamin D levels and age at onset among Morrocan MS cases^16^. However, these samples were taken several years after onset and may be influenced by mechanisms of reverse causality (i.e., environmental or behavioral changes associated with disease progression).

These findings do not fundamentally dismiss the role of vitamin D in MS onset nor the efficacy of vitamin D supplementation. Measures at symptomatic onset may not represent actual disease onset due to a likely period of sub-clinical disease activity. McDowell and colleagues observed that those reporting higher sun exposure or vitamin D supplementation during early adolescence (age 6–15 years old) had a delayed onset of disease of 2–4 years^17^. Therefore, the predictive value of circulating vitamin D and benefits of supplementation, if any, regarding the age of onset may only be relevant in early adolescence before symptomatic or perhaps even asymptomatic onset. However, a study examining lifelong residential UVB exposure observed no association to age at MS onset^18^. Genetic association studies and the recent popularity of genetic instruments may provide a reasonable proxy of vitamin D levels before onset. Still, recent studies observed no association between age at onset and genetic susceptibilities to vitamin D deficiency, further contradicting the role of vitamin D with age at MS onset^9,19^.

CSF IgG index was associated with age at onset, but this may be due to an association with age, as samples were taken near onset. IgG index have previously been shown to be negatively correlated with age among healthy individuals^20^. However, correlation with age was mainly attributed to a drop in IgG index above 45 years of age, and therefore unlikely to explain the association to age at onset as most patients in this cohort were below 45 years old. Our findings are also supported by an international multi-cohort study observing a similar negative correlation between IgG index and age at onset^21^. However, this association will require further validation in consideration of both age and disease duration.

Relapsing disease and sex were partly dependent on sampling time in this cohort resulting in a bias affecting the previously reported associations with serum vitamin-D levels^11^. Although this could be a random occurrence, annual fluctuations in vitamin D and sun exposure have been associated with relapse rate^22^, emphasizing the importance of correcting vitamin D measures for the time of sampling in future studies, particularly with a small sample size. It also showcases the effect of bias that occurs from non-randomized sampling where exploratory variables are confounded by variability in sampling protocol, in this case, resulting in a significant association in the opposite direction.

This study performs an exploratory re/examination of vitamin D and MS onset using reported data, and the interpretation of findings is limited in part from the reduced sample size. However, samples were taken at or near disease onset, limiting bias associated with disease progression prevalent in other studies. Unlike multicenter studies, sampling was performed locally, limiting environmental variability associated with measuring vitamin D.

In conclusion, these findings indicate serum vitamin D levels were not associated with age at onset, and that it is crucial to correct for seasonal variation of vitamin D to prevent misrepresented conclusions resulting from potential sampling bias. Further investigation and validation is required to understand the role of vitamin D during childhood/adolescence (i.e., before presymptomatic disease) and progression to symptomatic disease onset.

## Data Availability

Data used in this study have already been made publicly available.

## Abbreviations

25(OH)D: 25-Hydroxyvitamin D_3_
CSF: Cerebrospinal fluid
EDSS: Expanded disability status scale
MS: Multiple sclerosis
OCB: Oligoclonal band
PPMS: Primary progressive multiple sclerosis
RRMS: Relapsing-remitting multiple sclerosis
UV: Ultraviolet

## Code availability

Analytical scripts are available upon request.
